# Biological Performance of Primary Dental Pulp Stem Cells Treated with Gold Nanoparticles

**DOI:** 10.3390/biomedicines11092490

**Published:** 2023-09-08

**Authors:** Duaa Abuarqoub, Nouf Mahmoud, Walhan Alshaer, Marwa Mohammad, Abed Alqader Ibrahim, Mairvat Al-Mrahleh, Mohammad Alnatour, Dana A. Alqudah, Ezaldeen Esawi, Abdalla Awidi

**Affiliations:** 1 Department of Pharmacology and Biomedical Sciences, Faculty of Pharmacy and Medical Sciences, University of Petra, Amman 11196, Jordan; marwamohammad285@gmail.com; 2 Cell Therapy Center, University of Jordan, Amman 11942, Jordan; walhan.alshaer@ju.edu.jo (W.A.); mmairvat@gmail.com (M.A.-M.); pharmd.dana.alqudah@gmail.com (D.A.A.); ezaldeenesawi@gmail.com (E.E.); 3 Faculty of Pharmacy, Al-Zaytoonah University of Jordan, Amman 11733, Jordan; nouf.mahmoud@zuj.edu.jo; 4 Department of Biomedical Sciences, College of Health Sciences, QU Health, Qatar University, Doha 2713, Qatar; 5 Department of Nanoscience, Joint School of Nanoscience and Nanoengineering, University of North Carolina at Greensboro, 2907 E. Gate City Blvd., Greensboro, NC 27401, USA; amibrahim@uncg.edu; 6 Department of Pharmaceutics and Pharmaceutical Technology, Faculty of Pharmacy and Medical Sciences, University of Petra, Amman 11196, Jordan; mohammad.alnatour@uop.edu.jo; 7 School of Medicine, University of Jordan, Amman 11942, Jordan; 8 Department of Internal Medicine, Hospital of Jordan University, Amman University, Amman 11942, Jordan

**Keywords:** stem cells, dental, nanomaterials, AuNPs, biocompatibility

## Abstract

Gold nanoparticles (AuNPs) are one of the most stable nanoparticles that have been prevalently used as examples for biological and biomedical applications. Herein, we evaluate the effect of AuNPs on the biological processes of dental pulp stem cells derived from exfoliated deciduous teeth (SHED). Two different shapes of PEGylated AuNPs, rods (AuNR-PEG) and spheres (AuNS-PEG), were prepared and characterized. SHED cells were treated with different concentrations of AuNR-PEG and AuNS-PEG to determine their effect on the stemness profile of stem cells (SCs), proliferation, cytotoxicity, cellular uptake, and reactive oxygen species (ROS), for cells cultured in media containing-fetal bovine serum (FBS) and serum-free media (SFM). Our results showed that both nanoparticle shapes maintained the expression profile of MSC surface markers. Moreover, AuNS-PEG showed a stimulatory effect on the proliferation rate and lower toxicity on SHED, compared to AuNR-PEG. Higher concentrations of 0.5–0.125 nM of AuNR-PEG have been demonstrated to cause more toxicity in cells. Additionally, cells treated with AuNPs and cultured in FBS showed a higher proliferative rate and lower toxicity when compared to the SFM. For cellular uptake, both AuNS-PEG and AuNR-PEG were uptaken by treated cells efficiently. However, cells cultured in SFM media showed a higher percentage of cellular uptake. For ROS, AuNR-PEG showed a significant reduction in ROS at lower concentrations (<0.03 nM), while AuNS-PEG did not show any significant difference compared to the control untreated cells. Thus, our results give evidence about the optimum concentration and shape of AuNPs that can be used for the differentiation of stem cells into specific cell lineages in tissue engineering and regenerative medicine.

## 1. Introduction

Regenerative medicine is a new promising therapy for a wide range of diseases, due to its effective therapeutic impact in clinical applications based on cell-based therapies. It integrates the sciences and engineering disciplines to regenerate and repair damaged tissues and organs as well as a generation of new organs inside the laboratory that can perform the same function as the real organs [1,2,3]. Stem-cell-based therapy is one of the most important disciplines of regenerative medicine. They possess many features that make them capable of treating a wide range of uncured diseases [4,5,6]. Thus, SCs are considered an attractive source and powerful tool for tissue engineering and regenerative medicine by having the capability to renew themselves and their differentiation potential into a very wide range of lineages; osteocytes, chondrocytes, adipocytes, and various ectodermic and endodermic cell types [7,8,9]. Though the differentiation process of stem cells is laborious, differentiation protocols have a number of limitations: (a) they require the preparation of a mixture of supplements including growth factors, (b) they have costly chemical processes, and (c) their mechanism of action is not completely elucidated. Therefore, scientists have oriented their research toward finding new technology that can be used to overcome conventional methods.

Nanotechnology is a powerful tool that can stimulate the proliferation and differentiation of stem cells by improving the efficiency of using the cells in therapeutic strategies, which can be applied clinically in regenerative medicine [10,11]. The nanomaterial applications in the stem cell field have been highlighted for their tracking and therapy roles. Many studies have focused on the relationship between stem cells and nanomaterials [12,13,14]. The presence of metallic nanoparticles (MNPs), in the range of 1–100 nm, was first reported in an aqueous solution by Michael Faraday [15,16]. In comparison to other metallic particles, gold nanoparticles (AuNPs) have been widely used in different applications due to their unique properties such as ease of synthesis, optical properties, and biocompatibility. Additionally, the size, surface, geometrical structure, and shape can be controlled. Thus, AuNPs are suitable for different applications and requirements [16,17].

The physical and chemical properties of AuNPs have a significant impact on many cellular processes and responses including cellular uptake, proliferation, differentiation, and cytotoxicity [14,18]. All these characteristics play an essential role in internalizing the particles inside the cell. Therefore, any variation in these characteristics would result in changing any of these processes [19,20,21,22]. Previous studies have shown the mechanism of interaction of MNPs with cell components, either with the membrane or the intracellular structures, in addition to their effect on signaling pathways. According to previous studies, endocytosis is considered the primary cellular uptake mechanism of AuNPs [23,24,25,26,27,28].

Regarding the unique properties of AuNPs, such as their low toxicity and biocompatibility, many studies have reported the role of AuNPs in modulating the proliferation and differentiation of stem cells. For osteogenic differentiation, the properties of AuNPs have shown a great impact on the potential of treated MSCs to differentiate into osteocytes in a size-dependent matter. Previous studies have reported that 70 nm rod-shape AuNPs have stimulated osteogenic differentiation, whereas 40 nm rod shape AuNPs have inhibited the differentiation of the treated bone-marrow derived stem cells (BM-MSCs) [20]. Moreover, AuNPs with sizes 30 and 50 nm have a stimulatory effect on osteogenic differentiation. Meanwhile smaller AuNPs with a size of 4 nm have an inhibitory effect on the osteogenic differentiation of MSC and a stimulatory effect on the regeneration of reactive oxygen species (ROS) [20,21].

Additionally, AuNPs combined with other delivery systems have shown a stimulatory effect on the osteogenic differentiation potential of BM-MSCs [29,30]. Treatments of DPSCs with AuNPs solely assessed their potential to differentiate into osteogenic lineage [31] or as nanocomposite 3D culture [32,33]. Similarly, AuNPs induced the osteogenic differentiation of dental stem cells derived from periodontal ligaments (PDLSCs) in a size-dependent manner [34,35,36]. For ASCs, AuNPs solely, or combined to other biomaterials, promoted the differentiation of MSCs toward the osteogenic lineage, regardless of its size [21,22,37,38].

Previous studies have reported the potential of AuNPs to induce the differentiation of stem cells, according to their size, shape, and surface functional group [39,40]. Therefore, in this study, we aim to explore the impact of using two different shapes of AuNPs coated with polyethylene glycol (PEG) on the cellular processes of dental stem cells isolated from exfoliated deciduous teeth (SHED).

## 2. Materials and Methods

### 2.1. Preparation of AuNRs and PEGylated AuNRs (AuNR-PEG)

Gold nanorods (AuNRs) were synthesized using two surfactants as described previously with some modifications [41]. Briefly, 0.60 mL of ice-cold sodium borohydride (0.010 M, NaBH_4_ 99%, Sigma Aldrich, Burlington, MA, USA) was added to a mixture of cetyltrimethylammonium bromide (0.20 M, CTAB 99%; Sigma Aldrich, Burlington, MA, USA) and chloroauric acid 99.9% (0.005 M, HAuCl_4_ 99%, Sigma Aldrich, Burlington, MA, USA) for seed synthesis, which was confirmed by the formation of a light brown-colored solution. Then, the growth solution was synthesized by mixing 18 mL of silver nitrate (4 mM, AgNO_3_, Sigma Aldrich Burlington, MA, USA) with a mixture of sodium oleate (NaOH, Sigma Aldrich, Burlington, MA, USA) and CTAB in 250 mL of hot water (∼50 °C). After that, 250 mL of HAuCl_4_ (1 mM) was added to the growth solution and stirred for 90 min until it turned into a colorless solution. A few drops of HCl 37 wt%, 0.25 mL of ascorbic acid (64 mM), and 0.8 mL of the seed solution were then added to the growth solution. The obtained colloidal solution was then incubated for 24–48 h at 30 °C to enhance the formation of the nanorods. The formed AuNR suspension was centrifuged twice, and the pellets were dispersed in Milli-Q water.

The obtained AuNPs were coated with methoxy-polyethylene glycol-thiol (m-PEG-SH, MW ∼2000 g mol^−1^, Sigma Aldrich, Burlington, MA, USA) to enhance their colloidal stability. A volume of 0.1 mL of a 15 mg/mL m-PEG-thiol solution was added to each 1.0 mL of twice-centrifuged AuNRs and stirred overnight and then centrifuged at 10,000 rpm for 10 min. The resultant PEGylated AuNP pellets were dispersed in ultrapure water.

### 2.2. Preparation of AuNS and PEGylated AuNS (AuNS-PEG)

To boiled aqueous solutions of HAuCl_4_ (100.0 mL, 0.25 mM), 5.0 mL aqueous solution of 10% (*w*/*w*) sodium citrate was added. The heating was maintained for 5 min until a deep ruby red colored solution appeared indicating the formation of nanoparticles. After cooling, the Cit-GNS solution was centrifuged at 10,000 rpm for 10 min and re-suspended in ultrapure water. The prepared AuNS were coated with m-PEG-thiol using the same procedure used for AuNR PEGylation. The prepared nanoparticles were characterized by UV-visible absorbance spectroscopy, DLS, TEM, and zeta potential as described previously [41].

### 2.3. Cell Culture

#### 2.3.1. Subjects and Samples

Primary teeth were collected from children aged 5, 6, and 7 years old.

This study was approved by the institutional review board (IRB) at the Cell Therapy Center/University of Jordan (IRB-CTC/1-2022-06).

#### 2.3.2. Isolation and Expansion of Dental Stem Cells

Stem cells were extracted from the remnant pulp of primary teeth as previously described [42]. Briefly, the pulp tissue was collected by using root canal file size 25 nm, and then divided into smaller pieces. The tissue fragments were then maintained in the culture medium of mesenchymal stem cells (MSCs); alpha MEM containing 10% FBS, 1% L-Glutamine, and 1% Pencillin/streptomycin (Gibco, Carlsbad, CA, USA), incubated at 37 °C in 5% CO_2_ incubator.

#### 2.3.3. Immunophenotyping (Flow Cytometry)

To explore the effect of AuNPs on the stemness characteristics of stem cells, the MSC expression profile of stem cells; CD90, CD44, CD105, and CD73 were tested before and after treatment with AuNPs by using flow cytometry. Finally, the samples were analyzed by using FACS DIVA software version 8, using BD FACS Canto II flow cytometer instrument (BD Biosciences, NJ, USA). Data interpretation was performed using Flowlogic software version 7.3 (Melbourne, Australia).

### 2.4. Cell Proliferation Assay (MTT)

A total number of 5 × 10^3^ SHED cells were seeded into a 96-well plate (SPL, Naechon-Myeon Korea) and allowed to attach for 24 h, then treated with different concentrations of AuNR-PEG and AuNS-PEG at different concentrations; 0.5, 0.25, 0.125, 0.0626, 0.03, 0.015, and 0.007 nM, and incubated for 72 h at 37 °C in a 5% CO_2_ incubator in two serum conditions FBS and SFM. After incubation, the old medium was replaced with 100 µL of fresh medium and 15 µL of MTT [3-[4,5-dimethyl-2ndiazolyl]-2,5-diphenyl-2H-tetrazolium bromide] (5 mg/mL) (Promega, Madison, WI, USA) was added to each well, and the plates were incubated at 37 °C for 3 h. The reaction was stopped by the addition of 50 µL/well DMSO. After 10 min, the optical density (O.D.) was measured at 570 nm using a Glomax plate reader (Promega, Madison, WI, USA). The experiment was performed in triplicate for three independent experiments.

### 2.5. Cytotoxicity Assay: Apoptosis

The apoptosis/necrosis assay was used to determine whether our treatments had any cytotoxic effect on the derived cells. First, 1 × 10^5^ cells of SHED were seeded into a 6-well tissue culture plate (SPL, Naechon-Myeon, Korea). Following that, cells were treated with different concentrations of AuNR-PEG and AuNS-PEG at different concentrations; 0.5, 0.25, 0.125, 0.0626, 0.03, 0.015, and 0.007 nM, for 72 h, in two different serum conditions

Cells were harvested and collected with Trypsin EDTA 1X x (Euroclone, USA), after 72 h of treatment. The cells were then washed with PBS (Euroclone, USA) and centrifuged at 300× *g* for 5 min. The cells were stained with Annexin V/PI stain by using an apoptosis kit (Invitrogen, USA). Finally, the samples were analyzed by FACS DIVA 7 software, bersion 8, FACS Canto II (BD, Biosciences, NJ, USA). The experiment was performed in triplicate for three independent experiments.

### 2.6. Cellular Uptake

#### 2.6.1. Inductively Coupled Plasma−Optical Emission Spectroscopy (ICP–OES)

A density of 2 × 10^6^ SHED cells was seeded per 75 cm^2^ flask in a cell culture medium until reaching 70% confluence. Then, the cells were treated with (0.5 nM) of PEG-AuNR and PEG-AuNS suspensions in the tissue culture medium with FBS and serum-free medium (SFM) was immediately applied to the cells and incubated for 6 h. The cells were trypsinized after two washing steps with PBS, centrifuged at 300× *g* for 30 min at 4 °C, and the obtained cell pellets were mixed with aqua regia (HNO_3_ and HCl; 1:3) in a water bath (70 °C) for 3 h. After that, the digested samples were diluted with Milli-Q water up to 4.0 mL and filtered by a 0.22 μm Teflon syringe filter. Using a validated analytical method, inductively coupled plasma−optical emission spectroscopy (ICP–OES), the concentration (mg/L) and percentage of the internalized gold into cells were quantified at a wavelength of 242.795 nm using a calibration curve of gold standard for ICP method (0.2–10.0 ppm). The experiment was performed intriplicate.

#### 2.6.2. Confocal Microscopy

SHED cells were seeded into 75 cm^2^ flasks at a density of 2 × 10^6^ cells per flask in a growth culture medium and allowed to attach for 24 h. Then, the cells were treated with PEG-AuNR or PEG-AuNS suspensions (0.5 nM) in the tissue culture medium containing FBS and serum-free medium (SFM) was immediately applied to the cells for 6 h. The media was discarded after 6 h of incubation, and the cells were washed with PBS. The cells were fixed with 10% PFA for 10 min. Then, the fixed cells were washed thrice with the washing buffer. After that, 4′,6-diamidino-2-phenylindole (DAPI) stain (Thermofisher, Waltham, MA, USA) was added to the cells and incubated for 5 min, followed by a washing step with PBS. Finally, coverslips were transferred onto glass slides loaded with one drop of mounting media (DAKO, Glostrup, Denmark). Confocal images were acquired via a laser scanning microscope 780 (Zeiss, Oberkochen, Germany). The objective used for acquiring the images was a Plan-Apochromat 63X/1.4 Oil DIC M27. The cells were imaged at excitation/emission wavelengths of 532 nm/750 nm for gold and 360 nm/460 nm for DAPI.

#### 2.6.3. Transmission Electron Microscopy (TEM)

SHED cells were seeded into 75 cm^2^ flasks at a density of 2 × 10^6^ cells per flask in a growth culture medium and allowed to attach for 24 h. Then, the cells were treated with PEG-AuNR or PEG-AuNR suspensions (0.5 nM) in the tissue culture medium without FBS and were immediately applied to the cells for 6 h. The media was discarded after 6 h of incubation, and the cells were washed with PBS and trypsinized, and then centrifuged at 300× *g* at 4 °C. The cell pellets were collected after removing the supernatant and fixed in glutaraldehyde solution (3%) and phosphate buffer (pH 7.4). The cell pellets were washed in PBS and fixed in a buffer solution of osmium tetraoxide for 2 h. After that, the pellets were dehydrated in ethanol solutions and kept overnight in 1:1 (*v*/*v*) epoxy/propylene oxide. Then, the pellets were embedded in epoxy resin, and 70 nm thin sections were obtained using an ultramicrotome. The sections were fixed onto TEM grids (Formvar-coated) and imaged by TEM (Fei, Eindhoven, The Netherland). Cells not exposed to any treatment were used as a negative control.

### 2.7. Reactive Oxygen Species (ROS)

To determine the effect of the derived NPs on the production of ROS, cells were treated with AuNR-PEG and AuNS-PEG for 24 h and treated with different concentrations of NPs. Following that, cells were harvested by trypsin EDTA1X (Euroclone, Viafigino, Italy), and all samples were prepared by using a total reactive oxygen species assay kit (Invitrogen, Thermofisher, Waltham, MA, USA). Finally, the samples were analyzed by using FACS DIVA software version 8, using BD FACS Canto II flow cytometer instrument (BD, NJ, USA). Data interpretation was performed by using Flowlogic software version 7.3 (Melbourne, Australia). This experiment was performed in triplicate.

## 3. Results

### 3.1. Characterization of AuNPs

In this study, AuNRs were synthesized using a mixture of surfactants (oleic acid and cetyltrimethylammonium bromide: CTAB), to reduce the concentration of CTAB in the nanorods solution and consequently reduce the toxicity of the nanorods. The obtained nanorods were functionalized later with thiolated PEG to enhance their colloidal stability and to further reduce the concentration of CTAB. Nanoparticles are commonly functionalized with amine or thiol-terminated ligands to provide colloidal stability by steric repulsion [43,44]. The affinity of gold nanoparticles towards thiolated ligands is very well-known due to the high affinity of gold through the strong bond Au–SH.

**Figure 1:**
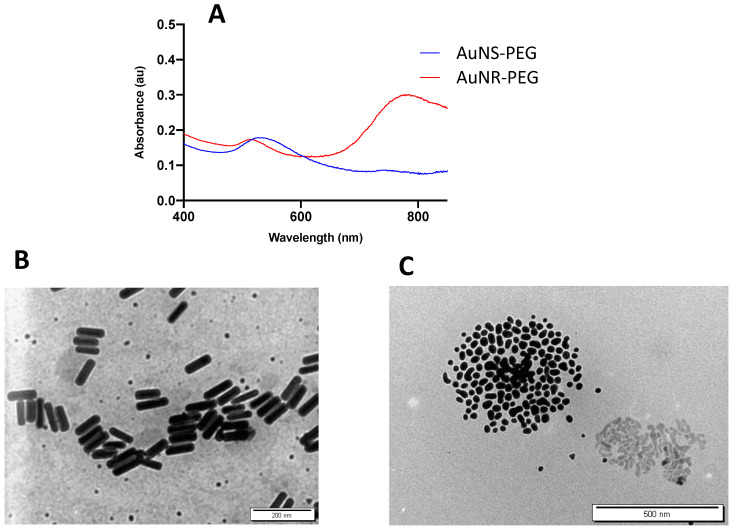
Optical absorbance and TEM images of AuNR–PEG (rod shape) and AuNS–PEG (spherical shape). (**A**) The optical absorbance spectra show a distinct peak for the nanosphere (AuNS–PEG) and two distinct peaks for nanorods (AuNR–PEG). TEM images confirm (**B**) the rod shapes (AuNR–PEG) (**C**) and spherical (AuNS–PEG) nanoparticles.

The optical spectrum of PEG–AuNRs demonstrated transverse and longintudinal peaks at ~530 and ~785 nm, respectively (Figure 1A). The peaks confirm the excellent colloidal stability of the nanorods where no broadening or tailing has been seen. The zeta potential of the PEGylated nanorods is around +2 which indicates the successful replacement of the positively charged surfactant (CTAB) by thiolated PEG molecules. The PEG ligands enhance the stability of the nanoparticles by steric repulsion between the PEG chains. The polydispersity index (PDI) is 0.205 for AuNS–PEG and 0.318 for AuNR–PEG.

The TEM images demonstrated the rod shape of the AuNRs with an average length, width, and aspect ratio of ~95 nm, ~25 nm, and 3.8, respectively (Figure 1B).

Regarding the AuNS, a distinct optical UV spectrum with a peak at ~530 nm Figure 1A was displayed. The zeta potential of the nanospheres is around neutral compared to the negatively charged citrate AuNS, which confirms the successful replacement of the citrate molecules with the thiolated PEG ligands. The TEM image shows spherical nanoparticles with an average particle size of ~34 nm (Figure 1C).

### 3.2. Expression of MSC Surface Markers

**Figure 2:**
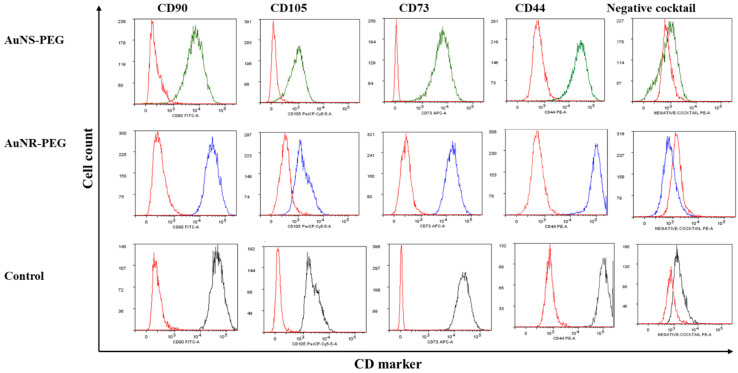
Flow cytometric histograms of the expression of stemness markers (MSC markers) of SHED cells treated with 0.5 nM of AuNS–PEG and AuNR–PEG AuNPs, for 24 h, and compared to the control untreated cells.

To determine whether AuNPs had any effect on the stemness properties of SHED cells, the surface markers of MSC were measured by flow cytometry. Our results showed that cells treated with 0.5 nM of either AuNR-PEG or AuNS-PEG have the same expression level of stemness markers (CD90, CD105, CD73, and CD44) and negative for hematopoietic stem cell markers (negative cocktail) as the control untreated cells. Figure 2 and Table 1.

### 3.3. Cell Proliferation Assay (MTT)

**Figure 3:**
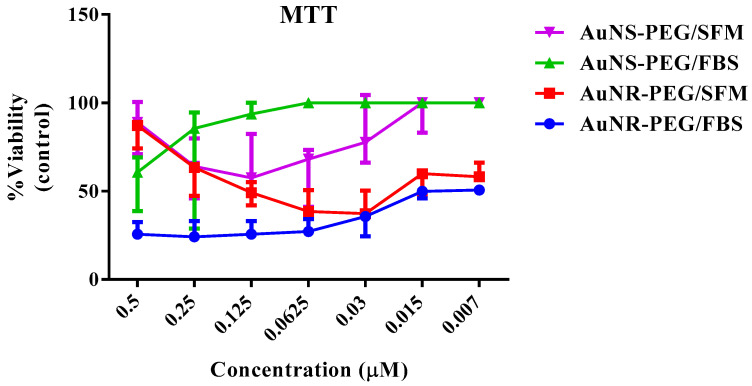
Cell proliferation assay (MTT) of SHED cells treated with different concentrations of AuNS–PEG and AuNR–PEG under two different serum conditions; fetal bovine serum (FBS) and serum-free medium (SFM) for 72 h, compared to the control untreated group from each condition. Data are presented as (Mean ± SD).

For the MTT assay, our results indicated that SHED cells treated with different concentrations of AuNR–PEG and AuNS–PEG showed a stimulatory effect on the growth rate of the derived cells while the concentrations were decreasing (*p <* 0.05). However, the cells treated with AuNS–PEG showed a significantly higher proliferative potential compared to cells treated with AuNR–PEG and their control untreated group (*p <* 0.05). Moreover, our results showed that cells cultured in FBS serum have shown the highest significant proliferation compared to other groups expanded in serum-free media (*p <* 0.05) as shown in Figure 3.

### 3.4. Cell Death Modality (Apoptosis/Necrosis)

**Figure 4:**
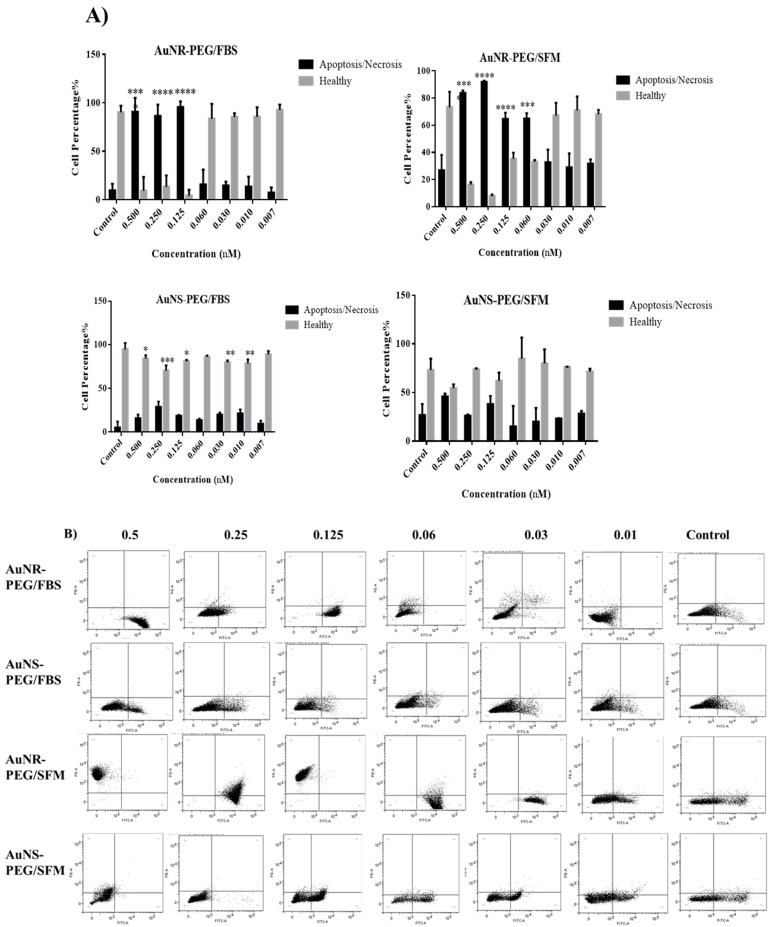
(**A**) Statistical analysis and (**B**) Flow cytometric dot plots, of cell death modality of SHED cells treated with different concentrations of GNR–PEG and GNS–PEG, cultured in FBS and SFM for 72 h. Data were analyzed by flow cytometry, representing the cell death modality: Early and late apoptosis, necrosis, and healthy cells. (* *p* < 0.05, ** *p* < 0.005, *** *p* = 0.0005, **** *p* < 0.0005).

Our results showed that AuNR–PEG NPs induced apoptotic cell death significantly (Figure 4) at high concentrations, 0.5–0.03 nM, while lower concentrations were nontoxic to the cells cultured to FBS when compared to the control untreated cells (*p* < 0.05). On the other hand, AuNS–PEG did not show any cytotoxic effect as the cells were healthy and did not show any cytotoxic effect when compared to the control untreated cells. Moreover, for the cells cultured in SFM, treated with either AuNR–PEG or AuNR–NS, the percentage of necrotic cells was increased when compared to the cells cultured in FBS media under the same conditions. However, the cells treated with AuNR–PEG and cultured in SFM showed the highest percentage of necrosis among all treatment groups. On the other hand, our results showed that cells treated with AuNS and SFM media showed a very low percentage of necrosis when compared to AuNR treated cells, as AuNS was less toxic and maintained the viability of cells in the absence of serum.

### 3.5. Cellular Uptake

#### 3.5.1. ICP–OES

Our results show that AuNR–PEG were up taken by treated cells at a significantly higher rate compared to the AuNS–PEG (*p <* 0.05). After 6 h of treatment for FBS and SFM cultured cells, AuNRs were 5- and 6-fold higher than cells treated with AuNS, respectively. Table 2.

#### 3.5.2. Imaging

##### Confocal Images

**Figure 5:**
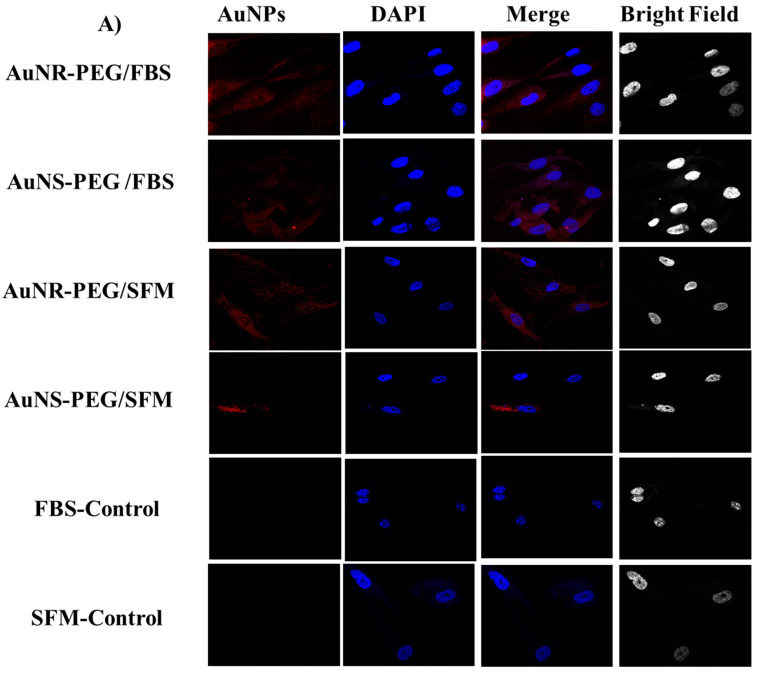

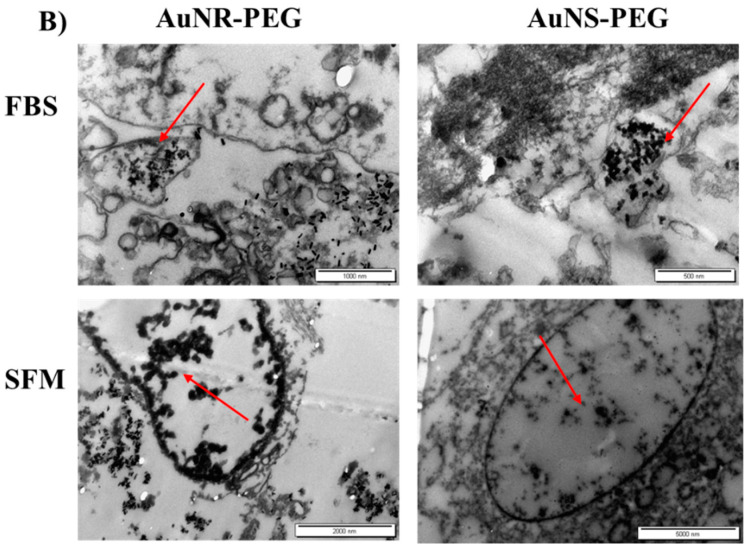
Cellular uptake images. (**A**) Confocal microscopy and (**B**) TEM images of SHED cells treated with 0.5 nM of AuNS–PEG and AuNR–PEG NPs and cultured in two different conditions, FBS and SFM, for 3 h. Red arrows showed AuNP particles inside the cell.

The cells treated with GNS–PEG and GNR–PEG were able to endocytose the NPs in both culture conditions; FBS and SFM. Figure 5A.

##### TEM

To confirm the uptake of gold nanoparticles by SHED cells, treated cells were examined by TEM. Our images showed that both AuNR–PEG and AuNS–PEG were accumulated inside the treated SHED cells in both culture conditions, FBs and SFM. Figure 5B show images of the spherical nanoparticles accumulated in the cells as aggregates compared to the nanorods which showed low aggregation tendency.

### 3.6. Reactive Oxygen Species (ROS)

**Figure 6:**
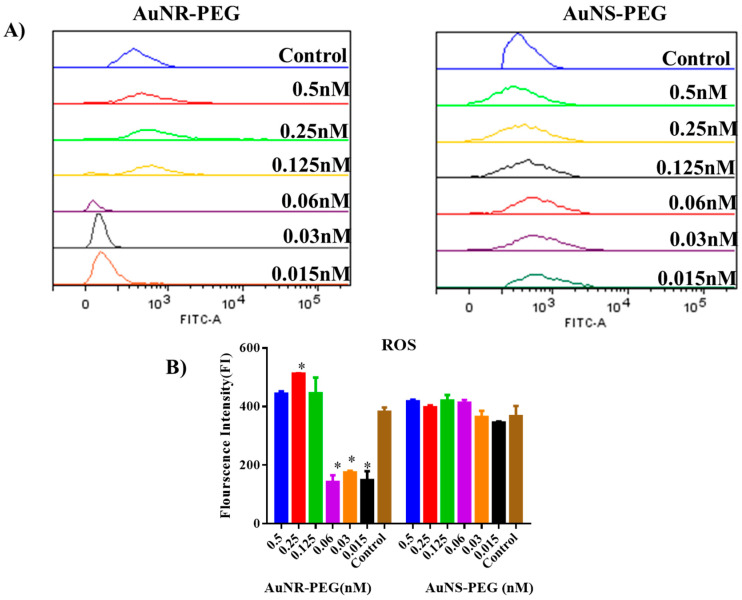
Reactive oxygen species (ROS). (**A**) Flow cytometric stacking histogram. (**B**) Statistical analysis for ROS released by SHED cells treated with different concentrations of GNR–PEG and GNS–PEG, for 24 h. Data are presented as (Mean ± SD). (* *p* < 0.05).

Our results indicated that AuNR–PEG were responsible for the increase in the secretion of ROS in cells treated with high concentrations above 0.03 nM, while lower concentrations decreased the release of ROS compared to the control untreated group significantly (*p* < 0.05). Meanwhile for AuNS–PEG, there was no stimulation for the production of ROS among the concentrations compared to the control untreated group. Figure 6.

## 4. Discussion

AuNPs have been widely used in different applications due to their unique properties such as ease of synthesis, optical properties, and biocompatibility. Additionally, the size, surface, geometry, and shape can be controlled toward specific applications and requirements [16,17]. Both physical and chemical properties of AuNPs have a great impact on many cellular processes such as cellular uptake, proliferation, differentiation, and cytotoxicity [13,16]. AuNPs have different sizes ranging from 4–100 nm with different surface chemical characteristics, resulting in variation in the final results. As AuNPs with sizes 30 and 50 nm have a stimulatory effect on osteogenic differentiation, the main aim of this study was to explore the impact of using two different shapes of AuNPs coated with polyethylene glycol (PEG) on the cellular processes of dental stem cells isolated from exfoliated deciduous teeth (SHED).

Our results showed that AuNS were more compatible and less toxic compared to AuNR. The AuNS increased the proliferation rate of the stem cells in a significant manner compared to the AuNRs treated group.

However, our data showed that the AuNPs showed negligible cytotoxicity to SHED at the studied Au concentrations of less than 0.25 nM. Moreover, the apoptosis assay showed that the concentration above 0.065 nM was toxic and showed high percentages of apoptotic and necrotic cell death. Both AuNS–PEG and AuNR–PEG were able to maintain the stemness properties of SHED. Interestingly, these results were similar to previously published research [21,45,46]. Additionally, for ROS production, cells treated with a lower concentration of GNR were able to reduce the production of ROS significantly after 24 h of treatment. These results were consistent with previously published data [47].

For the characterization of MSCs, both shapes of NPs were able to preserve the stemness of MSCs by maintaining the expression of stem cell surface markers, CD44, CD105, CD73, and CD90, when treating cells for a short period of time. However, previous studies have shown that the long-term culture of AuNPs will inhibit the expression of these markers by reducing the stemness state and enhancing their potential to differentiate [47].

Regarding cellular uptake, our quantitative results (ICP–OES) of AuNR were much higher when compared to AuNS. For confocal microscopy and TEM, the results showed that all the prepared NPs were up taken by stem cells. Our results were consistent with the previously published data [20,48,49]. Despite the presence of FBS protein, the cells were able to uptake the AuNPs efficiently. Moreover, the PEG increased the stability and functionality of the NPs. As was shown by Oberländer and his research group, the PEGylation of AuNPs is very crucial toward increasing the rate of uptake and decreasing the exocytosis of AuNPs from the cells. These data indicate the capability of NPs to achieve a higher loading efficiency, which reflects a long tracking procedure for stem cell differentiation [50]. However, for AuNS, the ICP–OES showed a low percentage of cellular uptake compared to AuNR. This could be explained by the fact that AuNS which are attached to the surface might be lost during the sample preparation of ICP–OES [50]. Furthermore, it is worth mentioning that nanoparticles–SFM have demonstrated less cellular uptake compared to those in FBS. These results are in good agreement with our previously published results where we found that the addition of (FBS) into the cell culture media significantly enhanced the colloidal stability by protein adsorption compared to those dispersed in serum-free media. Furthermore, nanoparticles dispersed in serum-free media showed a high rate and extent of cellular internalization compared to those dispersed in serum-containing media [44].

## 5. Conclusions

In the current study, the impact of two different shapes of the AuNPs (AuNS–PEG and AuNR–PEG) with various concentrations on the cellular processes of MSCs, cultured in two different serum conditions, FBS and SFM, were evaluated.

For cellular processes we studied the viability, cytotoxicity, and production of ROS at low concentrations (<0.0625 nM) of both AuNS and AuNR. They were more compatible and showed a stimulatory effect on the growth of treated cells and inhibitory impact on the secretion of reactive oxygen species (ROS). In addition, AuNS–PEG were more compatible in comparison to AuNR–PEG. For cellular uptake, the quantitative analysis showed that AuNR–PEG was more favorably uptaken by treated SHED cells. On the other hand, the surface modification of AuNPs (PEGylation) increased the stability of the AuNPs when culturing treated cells in media containing FBS. Thus, our results give evidence of the optimum concentration and shape of AuNPs that can be used in nanomaterials in stem cell therapeutic approaches, and which can be applied in tissue engineering and regenerative medicine.

## Figures and Tables

**Table 1:** The expression profile of stemness markers (MSC markers) of SHED cells treated with different concentrations of AuNR–PEG and AuNS–PEG.

	Concentration (nM)	CD90	CD105	CD73	CD44	Negative Cocktail
Control	0	99.94	58.22	99.95	90.49	0.17
AuNR–PEG-FBS	0.5	99.99	73.64	100	90.6	0.54
	0.250	98.99	18.16	99.57	99.8	0.14
	0.125	97.33	54.79	99.03	96.1	2.18
	0.6	100	25.14	99.91	100	97.9
	0.03	99.92	62.46	99.94	96.4	0.52
AuNS–PEG-FBS	0.5	99.86	89.21	99.71	99.14	0.36
	0.25	95.42	49.47	95.44	76.05	0.14
	0.125	95.58	45.48	96.09	90.66	0.05
	0.06	99.29	59.4	99.36	96.3	0.16
	0.03	98.86	59.96	98.93	95	0.44

**Table 2:** ICP–OES results of cells treated with 0.5 nM GNR–PEG and GNS–PEG, cultured in FBS and SFM media for 3 h.

Name	Conc (mg/L)	% Gold Uptake
AuNS–PEG–SFM	0.1408	0.556247
AuNR–PEG–SFM	6.004	23.71951
SFM-Control	Not detected	0
AuNS–PEG–FBS	0.0936	0.369778
AuNR–PEG–FBS	4.9884	19.70726
FBS-Control	Not detected	0

## Data Availability

The datasets used and/or analyzed during the current study areavailable from the corresponding author.
